# Assessment of aortic insufficiency during Impella support as a bridge to durable left ventricular assist device implantation and its long-term progression

**DOI:** 10.1007/s10047-026-01570-x

**Published:** 2026-07-04

**Authors:** Tomo Yoshizumi, Toru Kondo, Tomonari Uemura, Yasunari Hayashi, Shingo Kazama, Ryota Morimoto, Masato Mutsuga

**Affiliations:** 1 Department of Cardiac Surgery, Nagoya University Graduate School of Medicine, 65 Tsurumai-Cho, Showa-Ku, Nagoya, Aichi 466-8550 Japan; 2 Department of Cardiology, Nagoya University Graduate School of Medicine, 65 Tsurumai-Cho, Showa-Ku, Nagoya, Aichi 466-8550 Japan

**Keywords:** Impella, Left ventricular assist device, Aortic insufficiency, Mechanical circulatory support

## Abstract

Impella is increasingly used as a bridge to durable left ventricular assist device (dLVAD) implantation in patients with advanced heart failure. However, Impella support may worsen aortic insufficiency (AI), raising concerns regarding both AI progression during support and subsequent late AI progression after dLVAD implantation. This study evaluated the impact of Impella bridging on AI progression and clinical outcomes after dLVAD implantation. This retrospective single-center study included 64 patients who underwent primary dLVAD implantation and consisted of three analyses. First, baseline characteristics, perioperative variables, and early postoperative outcomes were compared between the aortic valve (AV) Intervention (n = 12) and No AV Intervention (n = 52) groups at dLVAD implantation. Second, changes in AI severity during Impella support were evaluated in patients with preoperative Impella support (n = 19). Third, long-term outcomes were compared between the Impella Bridging (n = 14) and No Impella Bridging (n = 38) groups after excluding patients who underwent concomitant AV intervention. Preoperative clinical severity was generally comparable between the AV Intervention and No AV Intervention groups, although hospital mortality was higher in the AV Intervention group. AI severity worsened significantly during Impella support (*p* < 0.001), whereas AV intervention rates did not differ significantly according to preoperative Impella support status (*p* = 0.32). During long-term follow-up, no statistically significant association was observed between Impella bridging and subsequent AI progression or clinical outcomes. Impella support as a bridge to dLVAD implantation was associated with significant worsening of AI during support. Under the current treatment strategy, including careful assessment of AV function and selective concomitant AV intervention at dLVAD implantation, no statistically significant association was observed between prior Impella bridging and subsequent late AI progression or adverse clinical outcomes. However, given the significantly higher in-hospital mortality in the AV Intervention group, careful patient selection for concomitant AV intervention remains essential.

## Introduction

Durable left ventricular assist device (dLVAD) implantation is an established therapeutic option for patients with advanced heart failure [1]; however, it is frequently complicated by late-onset aortic insufficiency (AI) [2, 3]. Truby et al. reported that the incidence of post-LVAD AI was 13.2%, and patients who developed moderate or greater AI had higher rates of rehospitalization for heart failure and decreased survival [2]. Surgical treatment for late AI after dLVAD implantation has been reported to be associated with a high incidence of right heart failure [4]; therefore, determining whether to perform prophylactic aortic valve (AV) intervention at the time of dLVAD implantation is clinically important.

With the expanding indications for dLVADs therapy, the use of the Impella (Abiomed, Danvers, MA, USA) as a bridge to dLVAD has attracted attention for its hemodynamic benefits [5]. However, Impella use is often associated with the development of AI, potentially caused by mechanical compression or trauma to the AV leaflets and root structures from the Impella catheter, as well as restricted leaflet mobility due to sustained valve closure [6, 7]. These mechanisms raise concerns that Impella bridging may increase long-term AI progression after dLVAD implantation, although definitive evidence remains lacking.

The aims of this study were threefold: (1) to evaluate the clinical characteristics and early outcomes associated with concomitant AV intervention at the time of primary dLVAD implantation; (2) to assess changes in AI severity during Impella bridging; and (3) to investigate long-term AI progression after dLVAD implantation in relation to Impella bridging.

## Methods

### Patients

This retrospective study included consecutive patients who underwent primary dLVAD implantation at Nagoya University Hospital between January 2018 and June 2025. The study protocol was approved by the Institutional Review Board of Nagoya University (protocol #2017-0281). Subsequent amendments were approved to cover the study period through June 2025. The requirement for written informed consent was waived because of the retrospective nature of the study. Instead, an opt-out approach was adopted through public disclosure of the study information on the institutional website, allowing patients to decline participation. The study was conducted in accordance with the principles of the Declaration of Helsinki.

### Data collection

Baseline demographic data (age, sex, body surface area, body mass index, etiology of heart failure, past medical history, and indication for dLVAD implantation) and clinical data (echocardiography, electrocardiogram, and laboratory findings) were collected at the latest time point before dLVAD implantation. Intraoperative data, including the type of dLVAD device, concomitant procedures, cardiopulmonary bypass (CPB) time, and total procedure time, were retrospectively obtained from the medical records.

## Study design and analytic frameworks

To evaluate changes in AI during Impella support and its impact on long-term AI progression after dLVAD implantation, we used three complementary analytic frameworks:

1. An AV intervention framework to evaluate the baseline characteristics and perioperative outcomes of patients who underwent concomitant AV intervention at the time of dLVAD implantation;
2. An Impella framework to assess changes in AI during Impella support; and
3. A dLVAD framework to assess the association between Impella bridging and long-term AI progression after dLVAD implantation.

### Evaluation of AI severity

AI severity was graded using an integrated semiquantitative echocardiographic assessment routinely used in our institutional practice, incorporating regurgitant jet extent, jet width-to-LVOT ratio, vena contracta width, and other standard parameters. AI severity was defined as follows: 0, none; 1, trivial; 2, mild or trivial–mild; 3, moderate or mild–moderate; and 4, severe or moderate–severe. For patients who required ongoing Impella support at the time of dLVAD implantation, AI severity was assessed intraoperatively using transesophageal echocardiography (TEE) after establishment of CPB and immediately following Impella removal. In patients who were not receiving Impella support at the time of surgery, AI severity was assessed preoperatively using transthoracic echocardiography (TTE). Long-term AI progression after dLVAD implantation was evaluated in patients who did not undergo concomitant AV intervention at the time of implantation.

### Surgical strategies for AI at the time of dLVAD implantation

The indication for concomitant AV intervention was primarily determined according to the severity of AI at the time of dLVAD implantation, while also considering patient condition and operative risk. From 2018 to 2021, AV intervention was generally indicated for moderate or greater AI, and either bioprosthetic AV replacement or the original Park’s stitch was performed. From 2022 onward, with the increasing number of patients undergoing implantation for destination therapy requiring long-term support, the threshold for intervention was lowered to mild or greater AI, and a modified Park’s stitch developed at our institution was used [8, 9].

### Patient management to prevent AI development after dLVAD implantation

All patients with dLVAD underwent monthly follow-up visits at the outpatient clinic. Hemodynamic parameters and the severity of AI were evaluated by both right heart catheterization and TTE at 3, 6, and 12 months, and annually thereafter following dLVAD implantation. Device speed optimization was performed based on these assessments. In addition, TTE alone was performed every six months.

### Statistical analysis

Continuous variables are presented as medians with interquartile ranges (IQRs), and categorical variables as frequencies and percentages. Between-group differences were assessed using the Mann–Whitney U test for continuous variables and Fisher’s exact test for categorical variables.

Changes in AI severity before and after Impella support were evaluated using the Wilcoxon signed-rank test. Long-term outcomes, including freedom from ≥ moderate AI, overall survival, and freedom from heart failure hospitalization, were assessed using Kaplan–Meier analysis with log-rank testing. Univariable Cox proportional hazards regression was used to evaluate the association between Impella bridging and long-term outcomes. Because of the limited number of events, multivariable Cox regression and propensity score–based approaches were not performed.

Restricted mean survival time (RMST) analyses up to 3 years after dLVAD implantation were additionally performed as complementary analyses that do not rely on the proportional hazards assumption. Differences in RMST between groups were estimated with 95% confidence intervals. E-values were calculated as sensitivity analyses for unmeasured confounding.

Two-tailed *p* values < 0.05 were considered statistically significant. All analyses were performed using JMP Student Edition 18 (SAS Institute Inc., Cary, NC, USA) and R version 4.6.0 (R Foundation for Statistical Computing, Vienna, Austria).

## Results

### Study population

**Fig. 1:**
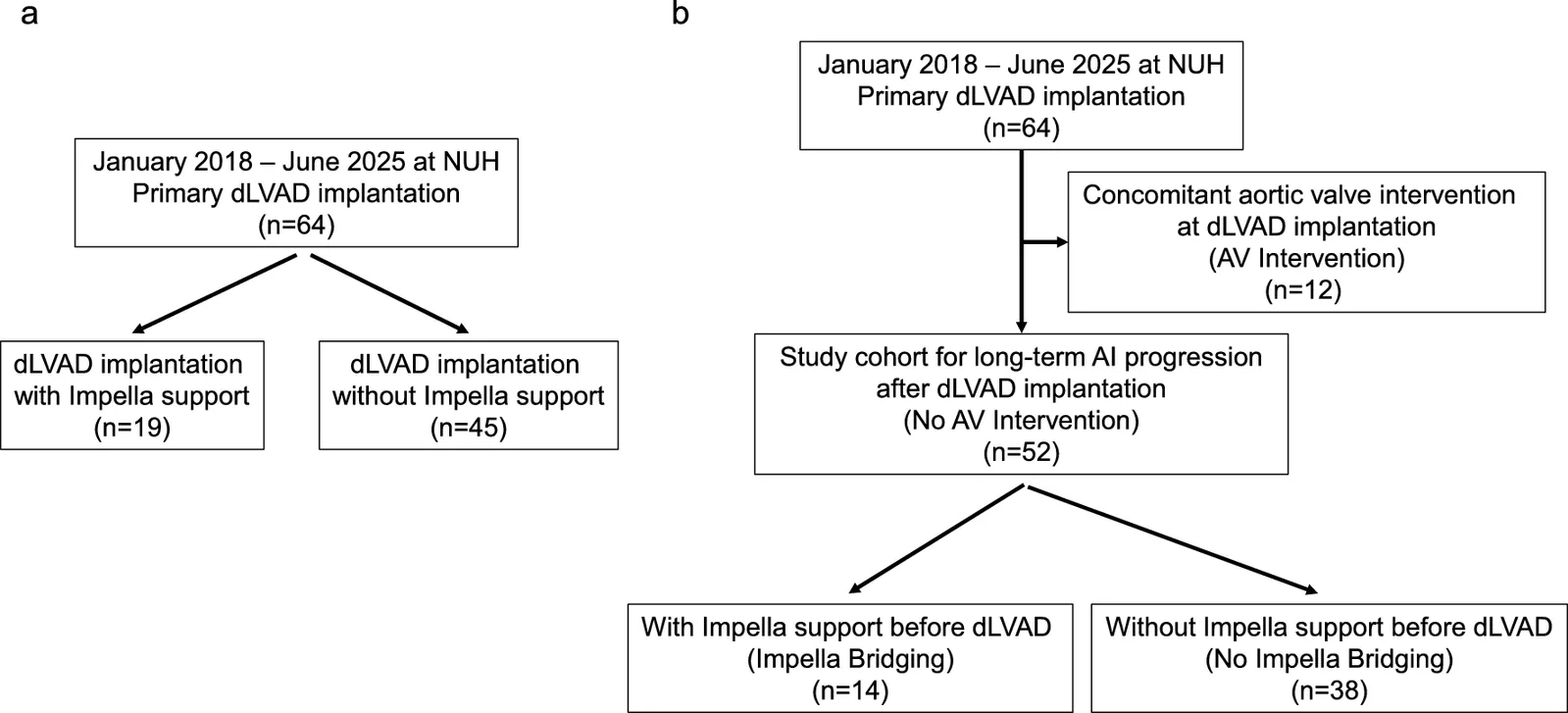
Study population and cohort selection. **a** Patients undergoing primary dLVAD implantation. **b** Cohort for long-term AI progression after excluding those with concomitant aortic valve intervention. The threshold for surgical AI treatment was ≥ moderate until 2021 and > mild from 2022. NUH, Nagoya University Hospital; dLVAD, durable left ventricular assist device; AI, aortic insufficiency

A total of 64 patients underwent primary dLVAD implantation, including 19 with Impella support and 45 without (Fig. 1a). Of these, 37 underwent dLVAD implantation between 2018 and 2021, and 27 from 2022 onward. Concomitant AV intervention was performed in six patients during each period (6/37 [16.2%] vs. 6/27 [22.2%]). After excluding patients with concomitant AV intervention, 52 patients were included in the long-term analysis (Fig. 1b). Concomitant AV procedures included central AV closure (original, n = 4; modified, n = 4), AV replacement (n = 3), and autologous pericardial valve closure (n = 1).

## Preoperative characteristics and perioperative outcomes according to concomitant AV intervention at dLVAD implantation

Several preoperative laboratory parameters and preoperative AI severity (*p* < 0.001) differed significantly between the AV intervention and No AV intervention groups. However, there were no significant differences in age, sex, INTERMACS profile, preoperative temporary MCS use, or comorbidities between the groups (Table 1).

**Table 1:** Preoperative characteristics according to concomitant AV intervention at dLVAD implantation

Variables	AV intervention (n = 12)	No AV intervention (n = 52)	*p* value
*Demographics*			
Age (median [IQR])	53 [42.8–59.8]	50 [42.8–58]	0.27
Male (%)	11 (91.7)	42 (80.8)	0.67
BSA (m^2^) (median [IQR])	1.58 [1.46–1.76]	1.62 [1.49–1.74]	0.47
BMI (kg/m^2^) (median [IQR])	20.41 [18.2–24.3]	19.9 [17.5–21.6]	0.16
Etiology			1.00
DCM (%)	8 (66.7)	32 (61.5)	
ICM (%)	1 (8.33)	7 (13.5)	
Others (%)	3 (25.0)	13 (25)	
INTERMACS			0.17
2 (%)	9 (75)	25 (54)	
3 (%)	3 (25)	22 (42)	
4 (%)	0	2 (4)	
DT (%)	3 (25)	9 (17.3)	0.68
*Comorbidities*			
Diabetes (%)	4 (33.3)	13 (25)	0.72
Hypertension (%)	3 (25)	5 (9.6)	0.16
Dyslipidemia (%)	4 (33.3)	14 (26.9)	0.73
Renal dysfunction (eGFR < 60 ml/min/1.73 m^2^) (%)	6 (50)	20 (38.5)	0.52
COPD (%)	1 (8.3)	1 (1.9)	0.34
Peripheral vessel disease (%)	1 (8.3)	1 (1.9)	0.34
Prior cardiac procedure (%)	3 (25)	9 (17.3)	0.68
History of CVA (%)	1 (8.3)	1 (1.9)	0.34
*Preoperative echocardiography*			
LVDd (mm) (median [IQR])	67.4 [56.3–77.4]	66 [58.7–76.0]	0.39
LVEF (%) (median [IQR])	17.9 [15.6–20.6]	17.6 [15.0–24.0]	0.40
AI severity at dLVAD implantation (%)			< 0.001
Grade 0	0	23 (44.2)	
Grade 1	0	23 (44.2)	
Grade 2	5 (41.7)	6 (11.6)	
Grade 3	6 (50)	0	
*Preoperative laboratory data*			
eGFR (ml/min/1.73 m^2^) (median [IQR])	62.6 [34.4–110.9]	70.3 [55.1–108.7]	0.16
BUN (mg/dL) (median [IQR])	22.5 [11.8–33.8]	15.4 [12.2–21.5]	0.11
Hgb (g/dL) (median [IQR])	10.3 [9.2–11.2]	11.6 [10.5–12.9]	0.007
T.Bil (mg/dL) (median [IQR])	1.3 [0.7–1.4]	1.0 [0.7–1.45]	0.04
Na (mmol/L (median [IQR]))	139 [135–141]	136 [133–138]	0.03
BNP (pg/mL) (median [IQR])	594 [230–1040]	456 [235–622]	0.10
*Preoperative temporary MCS before dLVAD implantation*			
Any MCS before dLVAD implantation (%)	9 (75)	27 (51.9)	0.20
Impella^*^	5 (41.7)	14 (26.9)	0.32
VA-ECMO	3 (25)	10 (19.2)	0.70
IABP	7 (58.3)	20 (38.5)	0.33
Extracorporeal LVAD	1 (8.3)	1 (1.9)	0.34
The duration of Impella support (days) (median [IQR])	44 [23.5–94.5]	24.5 [15.5–44]	0.12

^*^Patients receiving Impella concomitantly with other temporary MCS were categorized as Impella

Abbreviations: AV, aortic valve; AI, aortic insufficiency; dLVAD, durable left ventricular assist device; IQR, interquartile range; BSA, body surface area; BMI, body mass index; DCM, dilated cardiomyopathy; ICM, ischemic cardiomyopathy; DT, destination therapy; eGFR, estimated glomerular filtration rate; COPD, chronic obstructive pulmonary disease; CVA, cerebrovascular accident; LVDd, left ventricular end-diastolic diameter; LVEF, left ventricular ejection fraction; BUN, blood urea nitrogen; Hgb, hemoglobin; T.Bil, total bilirubin; BNP, B-type natriuretic peptide; MCS, mechanical circulatory support; VA-ECMO, venoarterial extracorporeal membrane oxygenation; IABP, intra-aortic balloon pump

Operative and early postoperative outcomes are summarized in Table 2. CPB time (*p* < 0.001) and operative time (*p* = 0.007) were significantly longer in the AV intervention group. No 30-day mortality was observed in either group, whereas in-hospital mortality was significantly higher in the AV intervention group (*p* < 0.001). Among the five in-hospital deaths in the AV intervention group, causes of death were intracranial hemorrhage (n = 2), multiple organ failure (n = 2), and infection (n = 1).

**Table 2:** Operative and early postoperative outcomes according to concomitant AV intervention at dLVAD Implantation

Variables	AV intervention (n = 12)	No AV intervention (n = 52)	*p* value
*Operative data*			
Type of durable LVAD			0.56
Heartmate II (%)	4 (33.3)	11 (21)	
Heartmate 3 (%)	8 (66.7)	40 (77)	
HVAD (%)	0	1 (2)	
Concomitant procedures (%)	12 (100)	46 (88.5)	0.58
CPB time (min)	171 [156.8–216.3]	134 [122.5–166.5]	< 0.001
Procedure time (min)	389 [326.8–517.8]	316.5 [273.5–356.5]	0.007
*Early postoperative outcomes*			
Hospital death (%)	5 (41.7)	0	< 0.001
30-Day mortality (%)	0	0	
ICU stay (days) (median [IQR])	11 [10–36.5]	11 [9.5–18.5]	0.28
Re-exploration for bleeding (%)	2 (16.7)	6 (11.5)	0.64
Stroke (%)	1 (8.3)	2 (3.8)	0.47
Septicemia (%)	4 (33.3)	2 (3.8)	0.009
Deep wound infection (%)	1 (8.3)	1 (1.9)	0.34
Prolonged ventilation (> 72 h) (%)	4 (33.3)	15 (28.8)	0.74
New postoperative dialysis (%)	3 (25)	4 (7.7)	0.12
Gastrointestinal complication (%)	4 (33.3)	4 (7.7)	0.03

Abbreviations: AV, aortic valve; dLVAD, durable left ventricular assist device; LVAD, left ventricular assist device; HVAD, HeartWare ventricular assist device; CPB, cardiopulmonary bypass; ICU, intensive care unit; IQR, interquartile range

### Development of AI during Impella support before dLVAD implantation

Among the 19 patients who received Impella support, the median support duration was 31 days (IQR: 16–47), and the median number of Impella devices used was 2 (IQR: 1–3). Concomitant AV intervention at dLVAD implantation was performed in 5 of 19 patients (26.3%) with Impella support and 7 of 45 patients (15.6%) without Impella support, with no significant difference between groups (*p* = 0.32).

**Fig. 2:**
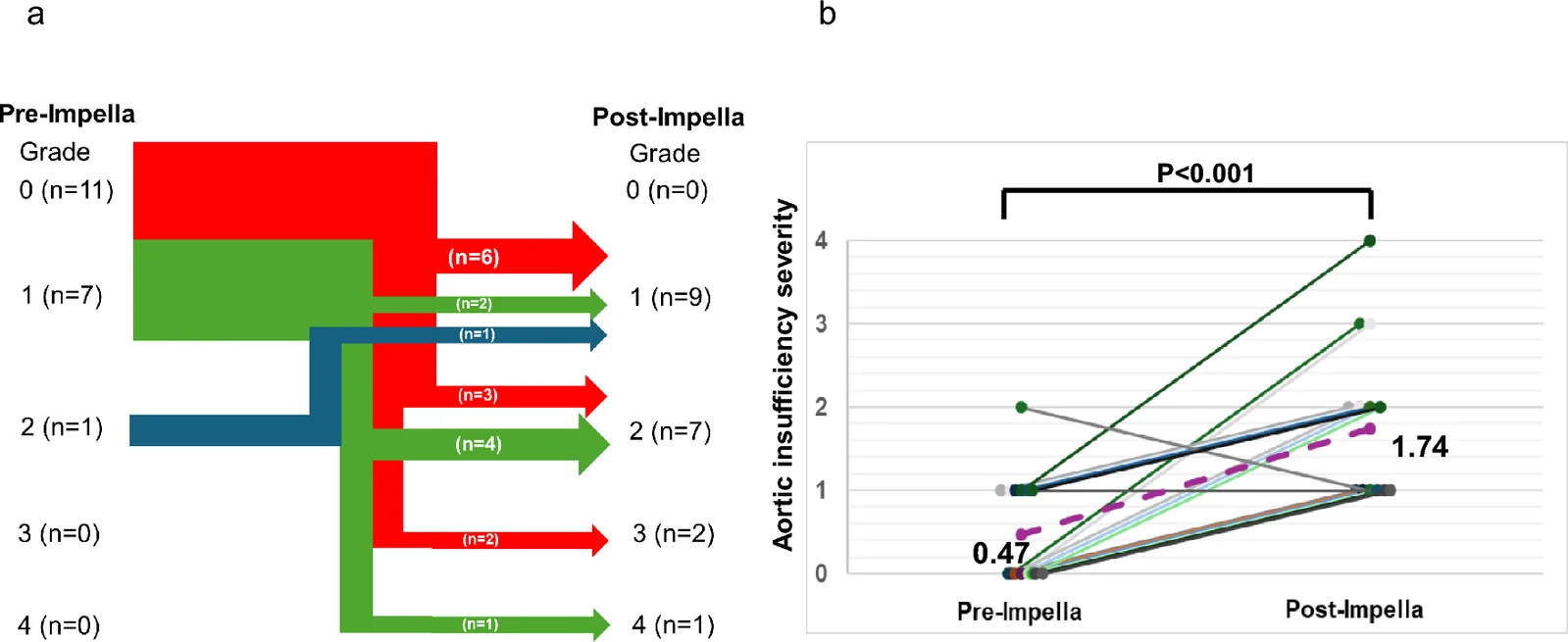
Changes in AI severity before and after Impella support. **a** Diagram showing transitions in AI severity from pre-Impella to post-Impella assessment. Colors indicate baseline AI grade (red, grade 0; green, grade 1; blue, grade 2). **b** Paired comparison of AI severity before and after support. Each line represents a patient. The purple dashed line indicates mean AI severity, increasing from 0.47 to 1.74 (*p* < 0.001). AI grades: 0, none; 1, trivial; 2, mild or trivial–mild; 3, moderate or mild–moderate; 4, severe or moderate–severe

Changes in AI severity before and after Impella support are illustrated by a diagram showing patient-level transitions in AI severity (Fig. 2a) and paired comparisons of individual patients (Fig. 2b). AI severity worsened significantly from 0.47 before Impella implantation to 1.74 after device removal (*p* < 0.001), and worsened in 16 of 19 patients (84%).

### Preoperative characteristics of patients undergoing dLVAD implantation without concomitant AV surgery

The 52 patients who did not undergo concomitant AV intervention at the time of dLVAD implantation were divided into the Impella Bridging group (n = 14) and the No Impella Bridging group (n = 38) according to preoperative Impella use, and preoperative characteristics were compared between groups (Table 3). The Impella Bridging group showed significant differences in INTERMACS profile (*p* < 0.001), AI severity at implantation (*p* < 0.001), preoperative laboratory data, and preoperative temporary MCS use (*p* < 0.001).

**Table 3:** Preoperative characteristics of patients without concomitant aortic valve intervention at primary dLVAD implantation

Variables	Overall (n = 52)	Impella bridging (n = 14)	No Impella bridging (n = 38)	*p* value
*Demographics*				
Age (median [IQR])	50 [42.8–58]	47.5 [36–54.3]	53 [42.8–59]	0.22
Male (%)	42 (80.8)	10 (71.4)	32 (84.2)	0.43
BSA (m^2^) (median [IQR])	1.62 [1.49–1.74]	1.54 [1.38–1.83]	1.63 [1.50–1.72]	0.22
BMI (kg/m^2^) (median [IQR])	19.9 [17.5–21.6]	20.99 [17.5–22.5]	19.5 [17.2–21.6]	0.54
Etiology				
DCM (%)	32 (61.5)	9 (64)	23 (61)	
ICM (%)	7 (13.5)	2 (14)	5 (13)	
Others (%)	13 (25)	3 (22)	10 (26)	
INTERMACS				< 0.001
2	25 (54)	14 (100)	14 (37)	
3	22 (42)	0	22 (58)	
4	2 (4)	0	2 (5)	
DT (%)	9 (17.3)	3 (21.4)	6 (15.8)	0.69
*Comorbidities*				
Diabetes (%)	13 (25)	4 (28.6)	9 (23.7)	0.73
Hypertension (%)	6 (9.6)	1 (7)	4 (10.5)	1.0
Dyslipidemia (%)	14 (26.9)	3 (21.4)	11 (29.0)	0.73
Renal dysfunction (eGFR < 60 ml/min/1.73 m^2^) (%)	20 (38.5)	3 (21.4)	17 (44.7)	0.2
COPD (%)	1 (1.9)	0	1 (2.63)	1.0
Peripheral vessel disease (%)	1 (1.9)	0	1	1.0
Prior cardiac procedure (%)	9 (17.3)	2 (14.3)	7 (18.4)	1.0
History of CVA (%)	1 (1.9)	1 (7.1)	0	0.27
*Preoperative echocardiography*				
LVDd (mm) (median [IQR])	66 [58.8–75.3]	67 [53.6–71.3]	65.4 [59.6–80.8]	0.53
LVEF (%) (median [IQR])	17.6 [15.0–24.0]	17.7 [16.3–21.1]	17.6 [14.7–25]	1.0
AI severity at dLVAD implantation (%)				< 0.001
Grade 0	23 (44.2)	0	23 (60.5)	
Grade 1	23 (44.2)	9 (64.3)	14 (36.8)	
Grade 2	6 (11.6)	5 (35.7)	1 (2.7)	
*Preoperative laboratory data*				
eGFR (ml/min/1.73 m^2^) (median [IQR])	70.3 [55.3–108.1]	122.3 [64.6–131.5]	66.8 [54.5–87.7]	0.06
BUN (mg/dL) (median [IQR])	15.4 [12.2–21.5]	14.9 [9.6–31]	16.4 [12.6–21.5]	0.21
Hgb (g/dL) (median [IQR])	11.6 [10.5–12.9]	10 [9.28–10.6]	12.3 [11.0–13.2]	< 0.001
T.Bil (mg/dL) (median [IQR])	1.0 [0.7–1.45]	1.25 [0.98–1.43]	0.8 [0.6–1.45]	0.04
Na (mmol/L (median [IQR]))	136 [133–138]	136.5 [133.8–138.3]	136 [133–138]	0.71
BNP (pg/mL) (median [IQR])	456 [235–622]	274 [230–590]	470 [251–668]	0.54
*Preoperative MCS*				
Temporary MCS at dLVAD implantation (%)				
No MCS	25 (48.0)	0	25 (65.8)	< 0.001
IABP	13 (25.0)	1 (7.1)	12 (31.6)	0.15
VA-ECMO	2 (3.7)	1 (7.1)	1 (2.6)	0.47
Impella^*^	10 (38.5)	10 (71.4)	0	< 0.001
Extracorporeal LVAD	1 (2.0)	1 (7.1)	0	0.27
Combination of MCS	1 (2.0)	1 (7.1)	0	0.27
The duration of Impella support (days) (median [IQR])	–	24.5 [15.5–44]	–	
The number of Impella for BTB (median [IQR])	–	2 [1–3]	–	

^*^Patients receiving Impella concomitantly with other temporary MCS were categorized as Impella

AI, aortic insufficiency; BMI, body mass index; BNP, B-type natriuretic peptide; BSA, body surface area; BTB, bridge-to-bridge; BUN, blood urea nitrogen; COPD, chronic obstructive pulmonary disease; CVA, cerebrovascular accident; DCM, dilated cardiomyopathy; dLVAD, durable left ventricular assist device; DT, destination therapy; eGFR, estimated glomerular filtration rate; Hgb, hemoglobin; IABP, intra-aortic balloon pump; ICM, ischemic cardiomyopathy; INTERMACS, Interagency Registry for Mechanically Assisted Circulatory Support; IQR, interquartile range; LVDd, left ventricular end-diastolic diameter; LVEF, left ventricular ejection fraction; MCS, mechanical circulatory support; Na, sodium; T.Bil, total bilirubin; VA-ECMO, venoarterial extracorporeal membrane oxygenation

**Fig. 3:**
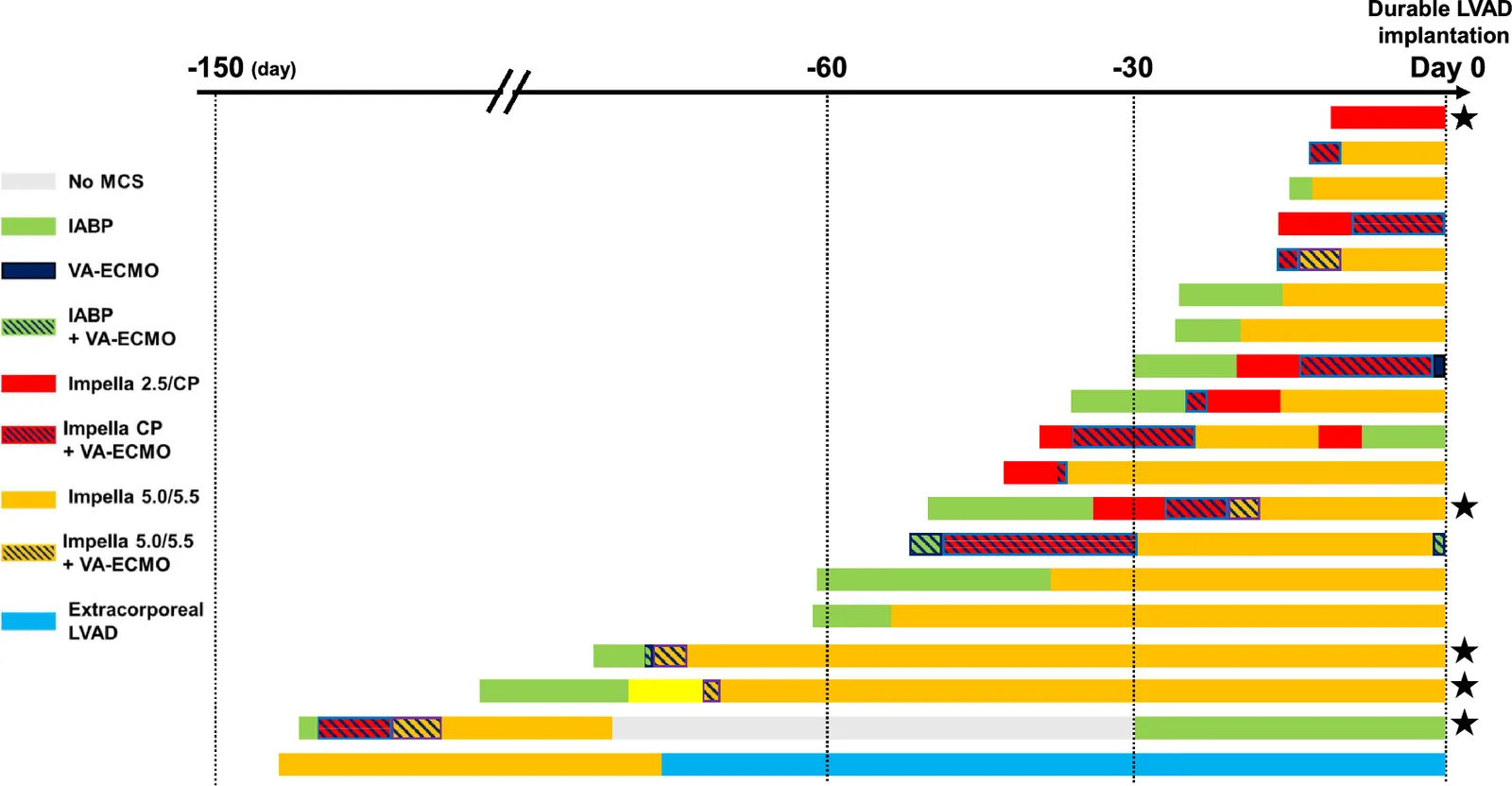
Swimmer plot of mechanical circulatory support trajectories in patients with preoperative Impella support. LVAD, left ventricular assist device; IABP, intra-aortic balloon pump; VA-ECMO, venoarterial extracorporeal membrane oxygenation. Each horizontal bar represents an individual patient. Colors and patterns indicate the type of temporary MCS support. Day 0 indicates the date of dLVAD implantation. The star symbol indicates concomitant aortic valve intervention at the time of dLVAD implantation. The time axis was truncated to improve visualization of support duration

Among patients who received Impella support, the median duration of support was 24.5 days (IQR: 15.5–44), with a median of 2 devices used (IQR: 1–3). All patients in the Impella Bridging group required sequential use of multiple temporary MCS devices before dLVAD implantation (Fig. 3).

### Intraoperative variables and postoperative in-hospital outcomes after dLVAD implantation without concomitant AV surgery

Intraoperative parameters and early postoperative outcomes were compared between the Impella Bridging and No Impella Bridging groups (Table 4). There were no significant differences in CPB or operative time between the two groups. No in-hospital mortality or significant differences in major postoperative complications were observed.

**Table 4:** Operative and early postoperative outcomes in patients without concomitant aortic valve intervention

	Overall (n = 52)	Impella bridging (n = 14)	No Impella bridging (n = 38)	*p* value
*Operative data*				
Type of durable LVAD (%)				
Heartmate II	11 (21)	2 (14)	9 (24)	0.71
Heartmate 3	40 (77)	12 (86)	28 (74)	0.48
HVAD	1 (2)	0	1 (2)	1.0
Concomitant procedures other than aortic valve surgery (%)	46 (88.5)	11 (78.6)	35 (92.1)	0.32
CPB time (mins)	134 [122.5–166.5]	158.5 [125.3–191.5]	131.5 [119.8–156.5]	0.11
Procedure time (mins)	316.5 [273.5–356.5]	350.5 [310.8–388]	309.5 [266.8–348.3]	0.22
*Early postoperative outcomes*				
Hospital death (%)	0	0	0	-
ICU stay (days) (median [IQR])	11 [9.5–18.5]	15.5 [10–30]	11 [8–16.25]	0.52
Re-exploration for bleeding (%)	6 (11.5)	1 (7.1)	5 (13.2)	1.0
Stroke (%)	2 (3.8)	1 (7.1)	1 (2.6)	0.47
Septicemia (%)	2 (3.8)	0	2 (5.3)	1.0
Deep wound infection (%)	1 (1.9)	0	1 (2.6)	1.0
Prolonged ventilation (> 72 h) (%)	15 (28.8)	7 (50)	8 (21.1)	0.08
New postoperative dialysis (%)	4 (7.7)	2 (14.3)	2 (5.3)	0.29
Gastrointestinal complication (%)	4 (7.7)	1 (7.1)	3 (7.9)	1.0

LVAD, left ventricular assist device; HVAD, HeartWare ventricular assist device; CPB, cardiopulmonary bypass; ICU, intensive care unit; IQR, interquartile range

### Long-term AI progression, survival, and heart failure hospitalization after dLVAD without concomitant AV intervention

All patients were followed up at our institution, with a median follow-up of 45.5 months (range, 1–84). Moderate or greater AI developed in 2 patients in the Impella Bridging group and in 12 patients in the No Impella Bridging group.

**Fig. 4:**
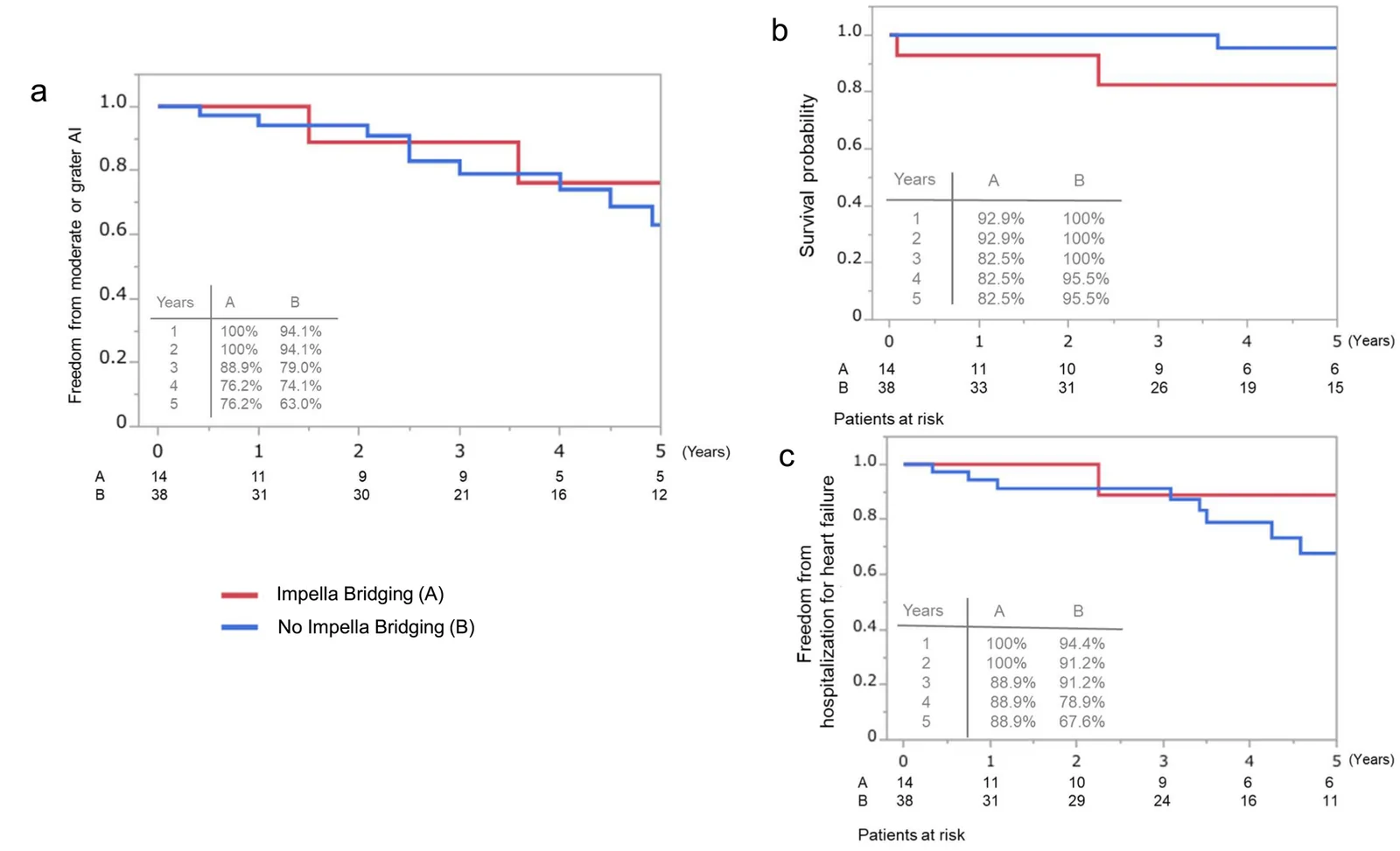
Clinical outcomes after durable LVAD implantation in patients without concomitant aortic valve intervention. Kaplan–Meier analyses comparing the Impella Bridging (A) and No Impella Bridging (B) groups for **a** freedom from moderate or greater aortic insufficiency, **b** overall survival, and **c** freedom from hospitalization for heart failure after durable LVAD implantation. Moderate or greater aortic insufficiency was defined as an aortic insufficiency grade ≥ 3. *p* values were calculated using the log-rank test

Both patients in the Impella Bridging group who developed moderate or greater AI had no AI before Impella insertion but developed trace or mild AI at the time of Impella removal and dLVAD implantation, followed by subsequent progression during follow-up. One patient developed moderate AI 43 months after dLVAD implantation and subsequently underwent heart transplantation. The other developed moderate AI at 18 months, which progressed to severe AI associated with continuous AV closure and resulted in heart failure hospitalization. Although surgical intervention was planned, the patient died suddenly, and autopsy revealed external outflow graft obstruction. Freedom from moderate or greater AI did not differ significantly between the groups (Fig. 4a and Table 5).

**Table 5:** Long-term outcomes after durable LVAD implantation according to preoperative Impella bridging

Outcome	HR	95% CI	*p* value	3-year RMST difference (months)	95% CI	*p* value	E-value
Freedom from ≥ moderate AI	1.36	0.64–2.87	0.849	0.45	− 3.91 to 4.79	0.841	2.06
Overall survival	0.84	0.60–1.20	0.312	− 3.33	− 8.17 to 1.52	0.179	1.67
Freedom from HF hospitalization	1.68	0.72–7.25	0.263	1.38	− 1.80 to 4.57	0.394	2.75

AI, aortic insufficiency; CI, confidence interval; HF, heart failure; HR, hazard ratio; RMST, restricted mean survival time

There were two deaths in each group, and overall survival did not differ significantly between the groups (Fig. 4b and Table 5).

Heart failure–related events occurred in 1 patient in the Impella Bridging group and in 8 patients in the No Impella Bridging group. Freedom from heart failure hospitalization did not differ significantly between the groups (Fig. 4c and Table 5).

## Discussion

In this study, AI severity worsened significantly during Impella support used as a bridge to dLVAD implantation. However, prior Impella support was not strongly associated with subsequent long-term AI progression after dLVAD implantation. In contrast, patients who underwent concomitant AV intervention had greater operative burden and higher in-hospital mortality. These findings suggest that concomitant AV intervention during Impella bridging should be determined based on both AI severity and operative risk.

### Concomitant AV intervention at dLVAD implantation

The 2023 ISHLT Guidelines for Mechanical Circulatory Support recommend surgical treatment for more than mild AI at the time of dLVAD implantation; however, the level of evidence remains limited (Class I, Level C) [10]. Tanaka et al. demonstrated that uncorrected mild AI at LVAD implantation was associated with subsequent AI progression and heart failure readmission [11]. However, that study did not evaluate patients undergoing concomitant AV intervention at implantation, and the optimal indications for aggressive intervention remain unclear.

In our institution, the surgical strategy for AI evolved during the study period in accordance with emerging guideline recommendations. In the present study, patients undergoing concomitant AV intervention had greater operative burden and significantly higher in-hospital mortality than those without AV intervention. However, given the observational design, limited sample size, and heterogeneous causes of death, no causal relationship between concomitant AV intervention and mortality can be inferred. Further studies are needed to better define the optimal indications for concomitant AV intervention during dLVAD implantation.

### Change of AI during Impella support as a bridge to dLVAD

Previous reports have described new-onset AI during Impella support, although clear risk factors remain unidentified [6, 7, 12, 13]. Proposed mechanisms include mechanical injury caused by the device and valvular remodeling associated with sustained AV closure during left ventricular unloading.

In the present cohort, 5 of 19 patients (26.3%) required concomitant AV intervention at the time of dLVAD implantation following Impella support. Intraoperative findings frequently showed degeneration or prolapse of the non-coronary cusp, possibly related to mechanical stress from the adjacent Impella catheter.

Among these 5 patients, Impella support exceeded 30 days in 4 patients, whereas 1 patient required intervention after only 11 days of support. Although prolonged Impella support may increase cumulative mechanical stress on the AV, these findings suggest that support duration alone is insufficient to determine the need for concomitant AV intervention. Therefore, careful reassessment of aortic valve function at the time of dLVAD implantation remains essential for surgical decision-making.

### Incidence and progression of AI after dLVAD implantation following Impella support

The incidence of moderate or greater AI during dLVAD support has been reported to range from 0 to 6% at 1 year and from 8 to 16% at 2 years [14, 15]. In the present study, freedom from moderate or greater AI was favorable and comparable to previous reports despite prior Impella support.

Impella support may predispose patients to subsequent AI progression through chronic mechanical stress on the AV. Higashi et al. demonstrated pathological AV injury after Impella 5.5 support, including inflammatory and interstitial cell infiltration, suggesting a potential mechanism for late AI development [6].

No clear association was observed between Impella bridging and long-term AI progression in Cox, RMST, or E-value analyses, although interpretation is limited by the small sample size and number of events.

In two cases of late-onset AI after Impella-bridged dLVAD implantation, regurgitant jets originated centrally without clear evidence of Impella-related injury. AI development in these cases was likely associated with leaflet and commissural degeneration caused by persistent AV closure and continuous-flow dLVAD support [14, 15].

Although Impella support may cause acute AV injury during bridging, prior Impella use was not clearly associated with subsequent long-term AI progression after dLVAD implantation in the present study, suggesting that late AI development during dLVAD support is likely multifactorial. However, these findings should be interpreted with caution because AI severity immediately after Impella removal was assessed under CPB, which may have underestimated the true magnitude of AI progression during Impella support. Nevertheless, under our current treatment strategy, which incorporates careful intraoperative reassessment of AV function together with selective concomitant AV intervention, prior Impella bridging was not clearly associated with subsequent long-term AI progression or adverse clinical outcomes.

### Clinical outcomes after dLVAD implantation following Impella support

Long-term outcomes after dLVAD implantation, including survival and freedom from heart failure hospitalization, were generally comparable to previous reports [1, 2]. Moderate or greater AI was observed in some patients who died, suggesting that late AI after dLVAD implantation may contribute to adverse outcomes [2].

In addition, moderate or greater AI frequently coexisted with ventricular arrhythmias (VA) in patients hospitalized for heart failure, with both present in approximately two-thirds of cases. VA may reduce cardiac output across the AV, leading to sustained valve closure and subsequent AI progression. Conversely, AI itself may promote LV distension and increase susceptibility to VA, thereby creating a vicious cycle that exacerbates heart failure.

The present findings suggest that Impella bridging itself may not be a major determinant of long-term clinical outcomes after dLVAD implantation.

### Comparison with previous studies

Several studies have evaluated AI progression after dLVAD implantation following Impella bridging. Rao et al. reported a higher incidence of mild or greater AI in patients supported with Impella, suggesting device-related AV injury or inflammation as potential mechanisms [16]. However, because pre-Impella echocardiographic data were unavailable in many patients, it remained unclear whether AI progression occurred during Impella support or after dLVAD implantation.

In contrast, Lewin et al. found no significant difference in moderate or greater AI between patients with and without preoperative Impella support [17], suggesting that persistent AV closure during continuous left ventricular unloading may play a more important role in late AI progression.

Late-onset AI patterns in the present cohort were generally consistent with previous reports. However, unlike previous studies, the present study evaluated both AI progression during Impella support and long-term outcomes after dLVAD implantation while also considering concomitant AV intervention. Favorable freedom from AI in our cohort may also be partly attributable to meticulous postoperative follow-up, including regular RAMP studies.

### Limitations

This study has several limitations. First, AI severity was assessed semi-quantitatively by echocardiography; however, the imaging modality and hemodynamic conditions differed according to perioperative status. In patients who required ongoing Impella support at the time of dLVAD implantation, AI severity was assessed intraoperatively using TEE after establishment of CPB and immediately following Impella removal, whereas other patients were evaluated preoperatively using TTE. Therefore, intraoperative assessment under CPB may have led to underestimation of AI severity because of altered loading conditions. Accordingly, the observed increase in AI severity during Impella support may have underestimated the true magnitude of AI progression.

Second, baseline differences between the Impella Bridging group and No Impella Bridging group limit direct comparisons and raise the possibility of residual confounding. In addition, temporary MCS strategies in the Impella Bridging group were heterogeneous, including device escalation and concomitant use of VA-ECMO or IABP, which may also have influenced subsequent outcomes. These factors should be considered when interpreting the study findings.

Third, indications for AV intervention evolved during the study period, which may have introduced temporal bias and affected comparisons between groups.

Finally, this was a single-center retrospective study with a small sample size and limited event numbers, precluding robust multivariable adjustment. Because the number of patients at risk became limited during later follow-up, RMST analyses were truncated at 3 years. Larger multicenter studies are needed to confirm these findings.

## Conclusions

Impella support as a bridge to dLVAD implantation was associated with significant worsening of AI during support. No statistically significant association was observed between prior Impella bridging and subsequent long-term AI progression or clinical outcomes. However, given the significantly higher in-hospital mortality observed in the AV Intervention group, careful patient selection for concomitant AV intervention at the time of dLVAD implantation remains essential.

## References

[1] Mehra MR, Goldstein DJ, Cleveland JC, Cowger JA, Hall S, Salerno CT, et al. Five-year outcomes in patients with fully magnetically levitated vs axial-flow left ventricular assist devices in the MOMENTUM 3 randomized trial. JAMA. 2022;328:1233–42. 36074476 10.1001/jama.2022.16197 PMC9459909

[2] Truby LK, Garan AR, Givens RC, Wayda B, Takeda K, Yuzefpolskaya M, et al. Aortic insufficiency during contemporary left ventricular assist device support: an analysis of the INTERMACS registry. JACC Heart Fail. 2018;6:951–60. 30384913 10.1016/j.jchf.2018.07.012 PMC6217859

[3] Uriel N, Milano C, Agarwal R, Lee S, Cleveland J, Goldstein D, et al. Incidence and clinical correlates of de novo aortic regurgitation with a fully magnetically levitated left ventricular assist device: a MOMENTUM 3 trial portfolio analysis. Eur J Heart Fail. 2023;25:286–94. 36404406 10.1002/ejhf.2746

[4] Gyoten T, Morshuis M, Fox H, Deutsch MA, Hakim-Meibodi K, Schramm R, et al. Secondary aortic valve replacement in continuous flow left ventricular assist device therapy. Artif Organs. 2021;45:736–41. 33432621 10.1111/aor.13906

[5] Saeed D, Feldman D, El Banayosy A, Birks E, Blume E, Cowger J, et al. The 2023 International Society for Heart and Lung Transplantation guidelines for mechanical circulatory support: a 10-year update. J Heart Lung Transplant. 2023;42:e1-222. 37245143 10.1016/j.healun.2022.12.004

[6] Chung JS, Emerson D, Ramzy D, Akhmerov A, Megna D, Esmailian F, et al. A new paradigm in mechanical circulatory support: 100-patient experience. Ann Thorac Surg. 2020;109:1370–7. 31563492 10.1016/j.athoracsur.2019.08.041

[7] Higashi H, Nishimura T, Aono J, Sakaue T, Kurata M, Izutani H, et al. Pathological evidence of native aortic valve injury after Impella support. Circ Heart Fail. 2021;14:e007571. 33478243 10.1161/CIRCHEARTFAILURE.120.007571

[8] Oishi H, Kondo T, Fujimoto K, Mutsuga M, Morimoto R, Hirano K-I, et al. Aortic insufficiency associated with Impella that required surgical intervention upon implantation of the durable left ventricular assist device. J Artif Organs. 2020;23:378–82. 32562105 10.1007/s10047-020-01184-x

[9] Park SJ, Liao KK, Segurola R, Madhu KP, Miller LW. Management of aortic insufficiency in patients with left ventricular assist devices: a simple coaptation stitch method (Park’s stitch). J Thorac Cardiovasc Surg. 2004;127:264–6. 14752440 10.1016/s0022-5223(03)01301-1

[10] Uemura T, Yoshizumi T, Hayashi Y, Kondo T, Morimoto R, Mutsuga M. Modified Park’s stitch using initial systematic cusp alignment in patients with left ventricular assist device. ASAIO J. 2025. 10.1097/MAT.0000000000002545. 40923628

[11] Tanaka Y, Nakajima T, Fischer I, Wan F, Kotkar K, Moon MR, et al. The impact of uncorrected mild aortic insufficiency at the time of left ventricular assist device implantation. J Thorac Cardiovasc Surg. 2020;160:1490-1500.e3. 32998831 10.1016/j.jtcvs.2020.02.144

[12] Butala B, Yu R, Schorr R, Gologorsky E. Periprocedural dynamics of aortic regurgitation in patients supported with an Impella left ventricular assist device. J Cardiothorac Vasc Anesth. 2020;34:659–62. 31668745 10.1053/j.jvca.2019.09.024

[13] Hironaka CE, Ortoleva J, Zhan Y, Chen FY, Couper GS, Kapur NK, et al. The effects of percutaneous left ventricular assist device placement on native valve competency. ASAIO J. 2022;68:541–6. 34419983 10.1097/MAT.0000000000001529

[14] Jimenez Contreras F, Mendiola Pla M, Schroder J, Bryner B, Agarwal R, Russell SD, et al. Progression of aortic valve insufficiency during centrifugal versus axial flow left ventricular assist device support. Eur J Cardiothorac Surg. 2022;61:1188–96. 35167677 10.1093/ejcts/ezac087

[15] Malick A, Ning Y, Kurlansky PA, Melehy A, Yuzefpolskaya M, Colombo PC, et al. Development of de novo aortic insufficiency in patients with HeartMate 3. Ann Thorac Surg. 2022;114:450–6. 34624263 10.1016/j.athoracsur.2021.08.074

[16] Rao SD, Johnson B, Olia SE, Wald J, Medina V, Rame JE, et al. Treatment with Impella increases the risk of de novo aortic insufficiency post left ventricular assist device implant. J Card Fail. 2020;26:870–5. 32681883 10.1016/j.cardfail.2020.06.014

[17] Lewin D, Rojas SV, Billion M, Meyer AL, Netuka I, Kooij J, et al. Durable left ventricular assist devices following temporary circulatory support on a microaxial flow pump with and without extracorporeal life support. JTCVS Open. 2024;21:168–79. 39534325 10.1016/j.xjon.2024.06.021 PMC11551302

